# LRRK2 is involved in the chemotaxis of neutrophils and differentiated HL-60 cells, and the inhibition of LRRK2 kinase activity increases *f*MLP-induced chemotactic activity

**DOI:** 10.1186/s12964-023-01305-y

**Published:** 2023-10-30

**Authors:** Yuichi Mazaki, Haruka Handa, Yoshizuki Fumoto, Takahiro Horinouchi, Yasuhito Onodera

**Affiliations:** 1 Department of Cellular Pharmacology, Graduate School of Medicine, Hokkaido University, Sapporo, Japan; 2 Department of Molecular Biology, Graduate School of Medicine, Hokkaido University, Sapporo, Japan; 3 Global Center for Biomedical Science and Engineering (GCB), Faculty of Medicine, Hokkaido University, Sapporo, Japan

**Keywords:** LRRK2, MFN2, Chemotaxis, Neutrophil, HL-60

## Abstract

**Background:**

Neutrophils depend heavily on glycolysis for energy production under normal conditions. In contrast, neutrophils require energy supplied by mitochondrial oxidative phosphorylation (OXPHOS) during chemotaxis. However, the mechanism by which the energy supply changes from glycolysis to OXPHOS remains unknown. Leucine-rich repeat kinase 2 (LRRK2) is partially present in the outer mitochondrial membrane fraction. *Lrrk2*-deficient cells show mitochondrial fragmentation and reduced OXPHOS activity. We have previously reported that mitofusin (MFN) 2 is involved in chemotaxis and OXPHOS activation upon chemoattractant *N*-formyl-Met-Leu-Phe (*f*MLP) stimulation in differentiated HL-60 (dHL-60) cells. It has been previously reported that LRRK2 binds to MFN2 and partially colocalizes with MFN2 at the mitochondrial membranes. This study investigated the involvement of LRRK2 in chemotaxis and MFN2 activation in neutrophils and dHL-60 cells.

**Methods:**

*Lrrk2* knockout neutrophils and *Lrrk2* knockdown dHL-60 cells were used to examine the possible involvement of LRRK2 in chemotaxis. *Lrrk2* knockdown dHL-60 cells were used a tetracycline-inducible small hairpin RNA (shRNA) system to minimize the effects of LRRK2 knockdown during cell culture. The relationship between LRRK2 and MFN2 was investigated by measuring the GTP-binding activity of MFN2 in *Lrrk2* knockdown dHL-60 cells. The effects of LRRK2 kinase activity on chemotaxis were examined using the LRRK2 kinase inhibitor MLi-2.

**Results:**

*f*MLP-induced chemotactic activity was reduced in *Lrrk2* knockout neutrophils in vitro and in vivo*. Lrrk2* knockdown in dHL-60 cells expressing *Lrrk2* shRNA also reduced *f*MLP-induced chemotactic activity. *Lrrk2* knockdown dHL-60 cells showed reduced OXPHOS activity and suppressed mitochondrial morphological change, similar to *Mfn2* knockdown dHL-60 cells. The amount of LRRK2 in the mitochondrial fraction and the GTP-binding activity of MFN2 increased upon *f*MLP stimulation, and the MFN2 GTP-binding activity was suppressed in *Lrrk2* knockdown dHL-60 cells. Furthermore, the kinase activity of LRRK2 and Ser935 phosphorylation of LRRK2 were reduced upon *f*MLP stimulation, and LRRK2 kinase inhibition by MLi-2 increased the migration to *f*MLP.

**Conclusions:**

LRRK2 is involved in neutrophil chemotaxis and the GTP-binding activity of MFN2 upon *f*MLP stimulation. On the other hand, the kinase activity of LRRK2 shows a negative regulatory effect on *f*MLP-induced chemotactic activity in dHL-60 cells.

Video Abstract

**Supplementary Information:**

The online version contains supplementary material available at 10.1186/s12964-023-01305-y.

## Background

Neutrophils are important innate immune cells that act as the first line of defense against pathogens. Most neutrophil chemoattractants, including the bacterial product *N*-formyl-Met-Leu-Phe (*f*MLP) and chemokines, bind to G protein-coupled receptors. Gßγ subunits, which released from heterotrimeric G protein, leads activation of various chemotaxis-related molecules including RAC1 and RAC2 through the activation of phosphoinositide 3‑kinase γ [1, 2]. During this process, the mitochondrial membrane potential of neutrophils increases after *f*MLP stimulation. Inhibition of mitochondrial oxidative phosphorylation (OXPHOS) suppresses the neutrophil chemotaxis [3, 4].

Mutations in Leucine-rich repeat kinase 2 (LRRK2) are a leading cause of familial Parkinson’s disease (PD) [5, 6]. LRRK2 is a large protein with two catalytic domains (GTPase and protein kinase) and multiple protein–protein interacting domains [7]. LRRK2 is present mainly in the cytoplasm, and approximately 10% is present in the mitochondrial fraction [8]. LRRK2 is associated with the outer mitochondrial membrane [9]. LRRK2 binds to mitofusin (MFN) 1, MFN2, optic atrophy 1 (OPA1), and dynamin-related protein 1 and partially colocalizes with MFN1, MFN2, and OPA1 at the mitochondrial membranes [10]. *Lrrk2* knockout cells show mitochondrial fragmentation and reduce OXPHOS activity and endoplasmic reticulum (ER)-mitochondrial interactions compared to wild-type cells [11, 12]. The kinase activity of LRRK2 is critical for mitochondrial morphology and function. The G2019S mutation in LRRK2 (LRRK2[G2019S]) is the most prevalent mutation in PD and leads to increased kinase activity of LRRK2 [13, 14]. LRRK2(G2019S)-expressing mouse embryonic fibroblasts (MEFs) showed mitochondrial fragmentation and reduced OXPHOS activity and ER-mitochondrial interactions compared to wild-type LRRK2 expressing MEFs [11]. In contrast, kinase-dead LRRK2(D1994A)-expressing MEFs showed increased of citrate synthase and OXPHOS activities compared to wild-type LRRK2 expressing MEFs [11].

MFN2 is a member of the dynamin superfamily and is mainly localized in the mitochondrial outer membrane [15]. MFN2 mediates mitochondrial outer membrane fusion and ER-mitochondria tethering via its GTPase activity [16]. Studies have reported that suppressing *Mfn2* expression using antisense RNA causes mitochondrial fragmentation and reduce OXPHOS activity [17].

We previously reported that *Mfn2* knockdown by small hairpin RNA (shRNA) suppresses mitochondrial morphological changes, OXPHOS, and chemotactic activity upon *f*MLP stimulation in differentiated HL-60 (dHL-60) cells [18]. Furthermore, *Mfn2* knockout neutrophils were reported to show reduced migration in zebrafish [19]. Here, we investigated whether LRRK2, which is reported to bind to MFN2, is involved in neutrophils chemotaxis. *Lrrk2* knockout neutrophils and *Lrrk2* knockdown dHL-60 cells were used to examine the relationship between LRRK2 and chemotaxis, and between LRRK2 and MFN2. These results suggest that LRRK2 is involved in neutrophil chemotaxis and is required for the GTP-binding activity of MFN2.

## Materials and methods

### Cell lines

HL-60 cells were purchased from American Type Culture Collection (Manassas, VA, USA). HL-60 cells were cultured in RPMI 1640 medium supplemented with L-glutamine (Nacalai Tesque, Kyoto, Japan) and 10% fetal bovine serum (Gibco, Billings, MT, USA). Cells were passaged once every two days. For differentiation into neutrophil-like cells, the cells were cultured in a medium supplemented with 1.25% dimethyl sulfoxide (DMSO; Merck, Rahway, NJ, USA) for 4 days.

### Animal models

C57BL/6-*Lrrk2*^*tm1.1Miff*^/J mice were purchased from Jackson Laboratory (Bar Harbor, ME, USA). All the mice were housed under standard laboratory conditions in a controlled pathogen-free room. Food and water were provided ad libitum. The protocols used for all animal experiments in this study were approved by Hokkaido University, Japan.

### Antibodies and chemicals

The following antibodies were used in this study: mouse monoclonal antibodies against ß-actin (Merck) and GAPDH (Santa Cruz Biotechnology, Santa Cruz, CA, USA), and rabbit monoclonal antibodies against MFN2 (Abcam, Tokyo, Japan), LRRK2 (Abcam), LRRK2 pS935 (Abcam), RAB10 (Abcam), RAB10 pT73 (Abcam), and TOMM20 (Cell Signaling Technology, Danvers, MA, USA). Horseradish peroxidase-conjugated anti-mouse and anti-rabbit donkey IgG antibodies were purchased from Jackson ImmunoResearch Laboratories (West Grove, PA, USA). Alexa Fluor 488-conjugated anti-rabbit goat IgG and Alexa Fluor 555-conjugated anti-mouse goat IgG antibody were purchased from Thermo Fisher Scientific (Waltham, MA, USA). Cis-2,6-dimethyl-4-(6-(5-(1-methylcyclopropoxy)-1H-indazol-3-yl)pyrimidin-4-yl)morpholine (MLi-2) was purchased from Cayman Chemical Co (Ann Arbor, MI, USA).

### Bone marrow neutrophils

Bone marrow neutrophils were prepared by centrifugation using Percoll gradients, as previously described [20]. Purified neutrophils were suspended in ice-cold Hank’s balanced salt solution (HBSS) containing 20 mM 4-(2-hydroxyethyl)-1-piperazineethanesulfonic acid (HEPES; pH 7.2) and 1% bovine serum albumin (BSA, Rockland Immunochemicals Pottstown, PA, USA) and stored on ice until further use.

### Gene silencing

LRRK2 was silenced by infecting HL-60 cells with lentiviruses constructed by inserting *Lrrk2* shRNA sequence (sh1: TRCN0000021462; sh2: TRCN0000021460, Merck) into Tet-pLKO-puro (Addgene, Watertown, MA, USA). Tet-pLKO-puro was a gift from Dr. Dmitri Wiederschain [21]. The targeting sequence of the irrelevant (Irr) shRNA has been previously reported [22]. The infected HL-60 cells were selected with 0.8 µg/ml puromycin (Merck). For gene silencing in dHL-60 cells, HL-60 cells expressing tetracycline-inducible *Lrrk2* shRNA or Irr shRNA were cultured in the presence of 1.25% DMSO for 1 day, followed by 3 days in a medium supplemented with 1.25% DMSO and 0.5 µg/ml doxycycline (Dox; FUJIFILM Wako Pure Chemical Corporation, Osaka, Japan).

### Chemotaxis assays

Transwell chemotaxis assays were performed as previously described [18, 23]. For neutrophils, transwell chemotaxis assays were performed using 24-well transwell chambers (pore size, 3.0 μm; Corning, Corning, NY, USA). For dHL-60 cells, transwell chemotaxis assays were performed using 24-well transwell chambers (pore size, 5.0 μm; Corning). Cells were placed on the upper side of the top chambers, and cells that migrated to the lower side of the chamber membrane were stained with Diff-Quick (Sysmex, Kobe, Japan) and counted. Five microscope fields of view were analyzed for each experiment.

For two-dimensional chemotaxis assays, a Dunn chamber (Hawksley, Sussex, UK) was used. After washing cells, the cells were attached to a coverslip and incubated at 37 °C for 10 min in HBSS containing 20 mM HEPES (pH 7.2) and 1% BSA. For neutrophils, coverslips were placed on a Dunn chamber, in which the outer well of the chamber was filled with the same solution supplemented with 10 µM *f*MLP (MP Biomedicals, Irvine, CA, USA) and a 1:10 dilution of 10% gelatin (Nacalai Tesque) in H_2_O. Cell migration was recorded by capturing images every 15 s for 20 min. For dHL-60 cells, the outer wells of the Dunn chambers were filled with the same solution, supplemented with 10 nM *f*MLP. Cell migration was recorded by capturing images every 15 s for 30 min. Image of each cell migration were recorded using an inverted microscope (Axiovert135, Carl Zeiss, Oberkochen, Germany) equipped with a digital camera (AxioCam and Axiovison software; Carl Zeiss). Stacks of images were then analyzed by ImageJ Fiji software (National Institutes of Health, Bethesda, MA, USA), with Chemotaxis and Migration Tool plug-in (Ibidi) for analysis of the cell tracking, velocity, and directional index of each moving cell. Cells that migrated more than 20 µm in their tracks during a 20 min incubation period were used for analysis.

### Air pouch analysis

Air pouch analysis of local inflammation was performed as described previously [24]. Mice were anesthetized by intraperitoneal injection of pentobarbital sodium and injected with 5 ml of air to create a subcutaneous dorsal pouch. After three days, the pouches were reinjected with 3 ml of air. Six days after the first injection, the pouches were injected with 1 ml of 0.5 μg/ml human interleukin-8 (IL-8) (Shenandoah Biotechnology, Warwick, PA, USA) and 0.5% carboxymethylcellulose (FUJIFILM Wako) in PBS. After 4 h, the mice were anesthetized, and the pouches were washed with 2 ml of PBS. The lavage fluid was immediately chilled on ice, the volume was recorded, and the neutrophils were counted.

### Immunofluorescence microscopy

Briefly, dHL-60 cells were attached to coverslips with HBSS containing 20 mM HEPES (pH 7.2) and 1% BSA for 10 min at 37 °C. After treatment with or without 10 nM *f*MLP for 5 min, the cells were fixed with 2% paraformaldehyde at 37 °C for 10 min, washed with PBS, and fixed with methanol at -20 °C for 5 min. The cells were then permeabilized with 0.1% Triton X-100 in PBS for 5 min, washed with PBS, and then incubated with 1% BSA in PBS for 30 min. The nuclei were stained with 4′,6-diamidino-2-phenylindole (DAPI; Nacalai Tesque). High-resolution structured illumination microscopy (SIM) images were acquired using an N-SIM microscope (Nikon, Tokyo, Japan) and NIS-elements software (Nikon), as previously described [18]. The percentage of cells with tubular mitochondria was calculated by counting cells with tubular mitochondria (length > 3 µm) and dividing this number by the total number of cells observed within each microscopic field, as described previously [18].

### Mitochondrial OXPHOS activity

The mitochondrial respiratory capacity was measured at 37 °C by using a high-resolution respirometer (Oxygraph-2 k, Oroboros Instruments, Innsbruck, Austria) as described previously [18]. Briefly, 2 × 10^6^ dHL-60 cells were added to the respirometer chamber filled with 2 ml of MiR05 medium (110 mM sucrose, 60 mM potassium lactobionate, 0.5 mM ethylene glycol tetraacetic acid, 3 mM MgCl_2_, 20 mM taurine, 10 mM KH_2_PO_4_, 20 mM HEPES [pH 7.1], and 1% BSA). The cells were then stimulated with 1 µM *f*MLP for 5 min and permeabilized with 5 µM digitonin (Merck). Substrates of OXPHOS and ADP were added to the respirometer chamber in the following order: (1) 2 mM malate, 10 mM glutamate, and 5 mM pyruvate (complex I-linked substrates); (2) 5 mM ADP + 3 mM MgCl_2_; and (3) 10 mM succinate (complex II-linked substrates). The O_2_ consumption rates (OCR) were expressed as O_2_ flux normalized to 1 × 10^6^ cells. Data acquisition and analysis of data were performed using DatLab software (Oroboros Instruments), as described previously [18].

### Mitochondrial fractionation

Mitochondrial fractionation was performed as previously described with some modifications [25]. dHL-60 cells were pre-incubated in HBSS containing 20 mM HEPES (pH 7.2) for 5 min at 37 °C and then stimulated with or without 1 µM *f*MLP at 37 °C for 5 min. The cells were lysed in hypotonic buffer (10 mM Tris-MOPS [pH7.4], 1 mM EDTA-Tris [pH7.4]) with rotation at 4 °C for 20 min. The lysed cells were homogenized by 15 repeated passes through a 30-gauge needle attached to a 1 ml syringe. The homogenate was centrifuged at 700 × *g* and 4 °C for 10 min, and the supernatant was centrifuged again at 700 × *g* and 4 °C for 10 min. The supernatant was then centrifuged at 7,000 × *g* and 4 °C for 10 min. The pellet was suspended with isotonic buffer (10 mM Tris-MOPS [pH7.4], 1 mM EDTA-Tris [pH7.4], 200 mM sucrose) and was centrifuged at 7,000 × *g* and 4 °C for 10 min. The pellets were again suspended with isotonic buffer and was centrifuged at 7,000 × *g* and 4 °C for 10 min. The pellets were resuspended in RIPA buffer (150 mM NaCl, 20 mM Tris–HCl [pH 7.4], 5 mM EDTA, 1% NP-40, 1% sodium deoxycholate, 0.1% SDS, 1 mM Na_3_VO_4_, 20 mM NaF, 10 mM sodium diphosphate, and a protease inhibitor cocktail for Use with Mammalian Cell and Tissue Extracts [Nacalai Tesque]) and incubated on ice for 10 min. The lysates were sonicated and centrifuged at 21,900 × *g* and 4 °C for 30 min. This supernatant was used as the mitochondrial fraction.

### GTP-binding assay

The GTP-binding assay for MFN2 was performed as previously described with some modifications [26]. dHL-60 cells were pre-incubated in HBSS containing 20 mM HEPES (pH 7.2) for 5 min at 37 °C, and then stimulated with or without 1 µM *f*MLP at 37 °C for 5 min. After stopping the reaction by adding ice-cold HBSS containing 20 mM HEPES (pH 7.2), the cells were centrifuged at 200 × *g* and 4 °C for 3 min. The cells were lysed in lysis buffer (150 mM NaCl, 50 mM Tris–HCl [pH 7.4], 1% Triton X-100, 5 mM MgOAc, 1 mM DTT, 1 mM Na_3_VO_4_, 20 mM NaF, 10 mM sodium diphosphate, and a protease inhibitor cocktail) on ice for 10 min. Cell lysates were sonicated and centrifuged at 21,900 × *g* and 4 °C for 10 min. After preclear, 250 µg cell lysates were incubated with GTP-agarose beads (Abcam) for 2 h at room temperature. The beads were then washed four times with lysis buffer, and proteins were eluted from the beads using the SDS sample buffer. The GTP-bound form of MFN2 was detected by western blotting with an anti-MFN2 antibody.

### Statistical analysis

All statistical analyses were performed using GraphPad Prism version.8 (GraphPad Software, Inc., Boston, MA, USA). Differences between multiple groups were calculated using one-way ANOVA followed by the Tukey–Kramer test. Differences between the two groups were calculated using a two-tailed Student’s *t*-test.

## Results

### *Lrrk2* knockout neutrophils show reduced *f*MLP-induced chemotactic activity

**Fig. 1:**
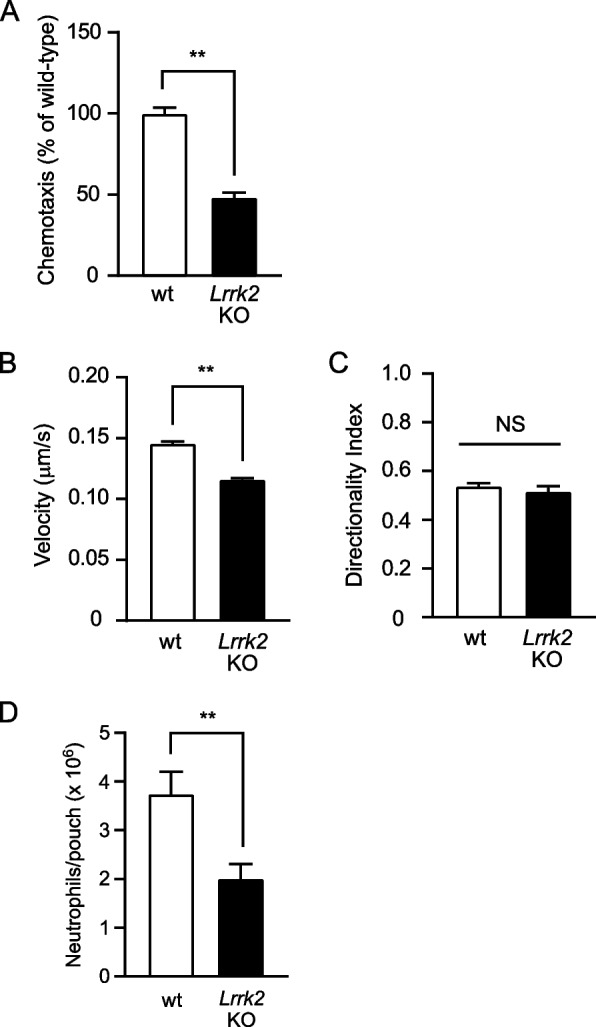
Inactivation of LRRK2 results in decreased neutrophil chemotactic activities. **A** Neutrophil chemotaxis assays in the transwell. Neutrophil migration in response to 10 µM *f*MLP was measured for 90 min in transwell chambers (*n* = 6 wells). The activity of wild-type neutrophils was taken as 100%. **B**, **C** Two-dimensional chemotaxis assays. Neutrophil migration in response to 10 µM *f*MLP was measured for 20 min in Dunn chambers (*n* > 45 cells). **B** Velocity. **C** Directionality index. **D** Air pouch analysis. Neutrophil numbers in exudates isolated 4 h after injection of IL-8 into subcutaneous dorsal air pouches (*n* = 10 mice). wt, wild-type mice. *Lrrk2* KO, *Lrrk2* knockout mice. Error bars, SEM. ** *p* < 0.01 compared with wild-type mice. NS, not significant

Previously, it was reported that *Lrrk2* knockout causes mitochondrial fragmentation and reduces OXPHOS activity [11, 12]. Since the inhibition of OXPHOS activity is reported to reduce neutrophil chemotactic activity, we first examined whether *Lrrk2* is involved in neutrophil chemotaxis using transwell chambers. The *f*MLP-induced chemotactic activity of *Lrrk2* knockout neutrophils was lower than that of wild-type neutrophils in transwell chamber assays (Fig. 1A). Next, we examined two-dimensional chemotaxis using Dunn chambers to assess the velocity and directionality. *Lrrk2* knockout neutrophils showed decreased velocity compared to wild-type neutrophils, whereas no change was observed in the directionality index of *Lrrk2* knockout neutrophils compared to that of wild-type neutrophils (Fig. 1B, C, S1, Additional files 1, 2). Furthermore, we examined the chemotaxis of *Lrrk2* knockout neutrophils in vivo using an air pouch model. The accumulation of neutrophils in an air-pouches during the 4 h interval after the injection of IL-8 into the air pouches was lower in *Lrrk2* knockout mice than in wild-type mice (Fig. 1D). These results suggest that LRRK2 is involved in neutrophil chemotaxis in vitro and in vivo.

### *Lrrk2* knockdown dHL-60 cells show reduced *f*MLP-induced chemotactic activity

**Fig. 2:**
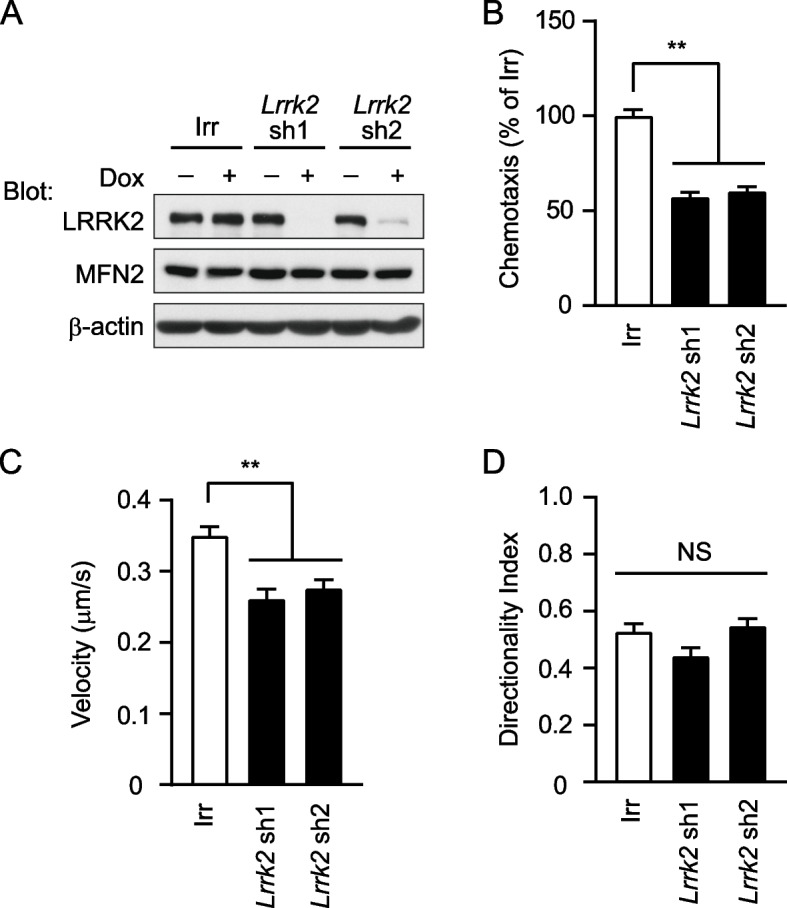
*Lrrk2* knockdown results in a decrease in the chemotactic activities of dHL-60 cells. **A** Expression pattern of proteins in *Lrrk2* knockdown dHL-60 cells. dHL-60 cells expressing tetracycline-inducible *Lrrk2* shRNA or irrelevant (Irr) shRNA were cultured in the presence of 1.25% DMSO with or without 0.5 µg/ml doxycycline (Dox) for 3 days, and analyzed for expression of the indicated proteins. **B** Transwell chemotaxis assays of dHL-60 cells. dHL-60 cells expressing tetracycline-inducible *Lrrk2 *shRNA or Irr shRNA were cultured with 0.5 µg/ml Dox for 3 days. dHL-60 cells migration in response to 10 nM *f*MLP was measured for 120 min in transwell chambers (*n* = 6 wells). The activity of Irr shRNA expressing dHL-60 cells was taken as 100%. **C**, **D** Two-dimensional chemotaxis assays. dHL-60 cells expressing tetracycline-inducible *Lrrk2* shRNA or Irr shRNA were cultured with 0.5 µg/ml Dox for 3 days. dHL-60 cells migration in response to 10 nM *f*MLP was measured for 30 min in Dunn chambers (*n* > 25 cells). **C** Velocity. **D** Directionality index. ** *p* < 0.01 compared with Irr shRNA expressing dHL-60 cells. NS, not significant

HL-60 cells are differentiated into a granulocytic lineage by DMSO and are used as a model for neutrophils [19, 27, 28]. To thoroughly investigate the role of LRRK2 in chemotaxis, we analyzed dHL-60 cells. We used a tetracycline-inducible system to minimize the effects of LRRK2 knockdown on cell growth during cell culture. Tetracycline-induced *Lrrk2* shRNA suppressed LRRK2 expression without altering MFN2 expression in dHL-60 cells (Fig. 2A). We then used transwell chambers to verify whether *Lrrk2* knockdown dHL-60 cells exhibited reduced chemotactic activity. *Lrrk2* knockdown dHL-60 cells showed reduced chemotactic activity compared to Irr shRNA-expressing dHL-60 cells (Fig. 2B). We found that *Lrrk2* knockdown in dHL-60 cells, like neutrophils, slowed without changing the directional index in a two-dimensional chemotaxis assay (Fig. 2C, D, Supplementary Figure S2, Additional files 3, 4, 5). In contrast, Irr shRNA-expressing and *Lrrk2* knockdown dHL-60 cells cultured without doxycycline showed no significant difference in chemotactic activity in the transwell chambers and two-dimensional chemotaxis assays (Supplementary Fig. S3). These results suggest that LRRK2 is involved in the chemotaxis of dHL-60 cells.

### Knockdown of *Lrrk2* reduces OXPHOS activity and suppresses mitochondrial morphological changes

**Fig. 3:**
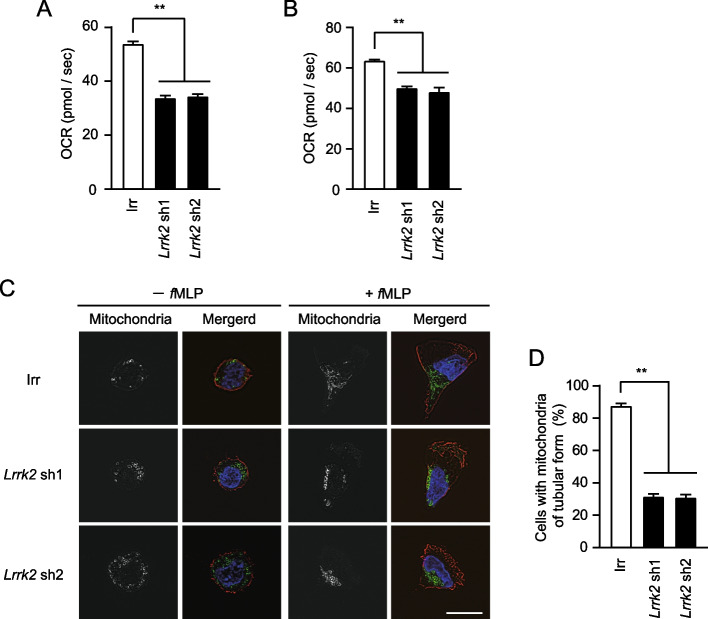
*Lrrk2* knockdown results in a decrease in OXPHOS activities of dHL-60 cells. **A**, **B** dHL-60 cells expressing tetracycline-inducible *Lrrk2* shRNA or irrelevant (Irr) shRNA were cultured in 1.25% DMSO with 0.5 µg/ml doxycycline (Dox) for 3 days. The OXPHOS activities of these cells were measured after treatment with 1 µM *f*MLP for 5 min (*n* = 4). Error bars, SEM. **A** complex I. **B** complex I + II. **C** Representative immunofluorescence microscope images of mitochondria. dHL-60 cells expressing tetracycline-inducible *Lrrk2* shRNA or irrelevant (Irr) shRNA were cultured in 1.25% DMSO with 0.5 µg/ml Dox for 3 days. dHL-60 cells attached to coverslips were then incubated with or without 10 nM *f*MLP for 5 min. Green; anti-TOMM20, Red; anti-ß-actin, Blue; DAPI. Bar, 10 µm. **D** Percentages of cells with mitochondria of tubular form after *f*MLP stimulation. > 50 cells were analyzed in four independent experiments. Error bars, SEM. ** *p* < 0.01 compared with Irr shRNA expressing dHL-60 cells

*Lrrk2* knockdown dHL-60 cells showed reduced *f*MLP-induced chemotactic activity, similar to *Mfn2* knockdown dHL-60 cells. Next, we examined whether *Lrrk2* knockdown dHL-60 cells showed a reduction in OXPHOS activity, similar to *Mfn2* knockdown dHL-60 cells. We found that OXPHOS activity at complex I and complex I + complex II was reduced in *Lrrk2* knockdown dHL-60 cells (Fig. 3A, B).

Immunofluorescence microscopy using an anti-TOMM20 antibody showed that the mitochondrial morphology changed from spherical to tubular upon *f*MLP stimulation in dHL-60 cells [18]. We examined whether *Lrrk2* knockdown dHL-60 cells suppressed mitochondrial morphological changes similar to *Mfn2* knockdown dHL-60 cells. In Irr shRNA-expressing dHL-60 cells, 87.8% of the transformed into cells with tubular mitochondria upon *f*MLP stimulation (Fig. 3C, D). In contrast, *Lrrk2* knockdown dHL-60 cells transformed into cells with tubular mitochondria at approximately 31% cells, suggesting that *Lrrk2* knockdown suppresses mitochondrial morphology changes upon *f*MLP stimulation in dHL-60 cells (Fig. 3C, D).

### LRRK2 affects the GTP-binding activity of MFN2

**Fig. 4:**
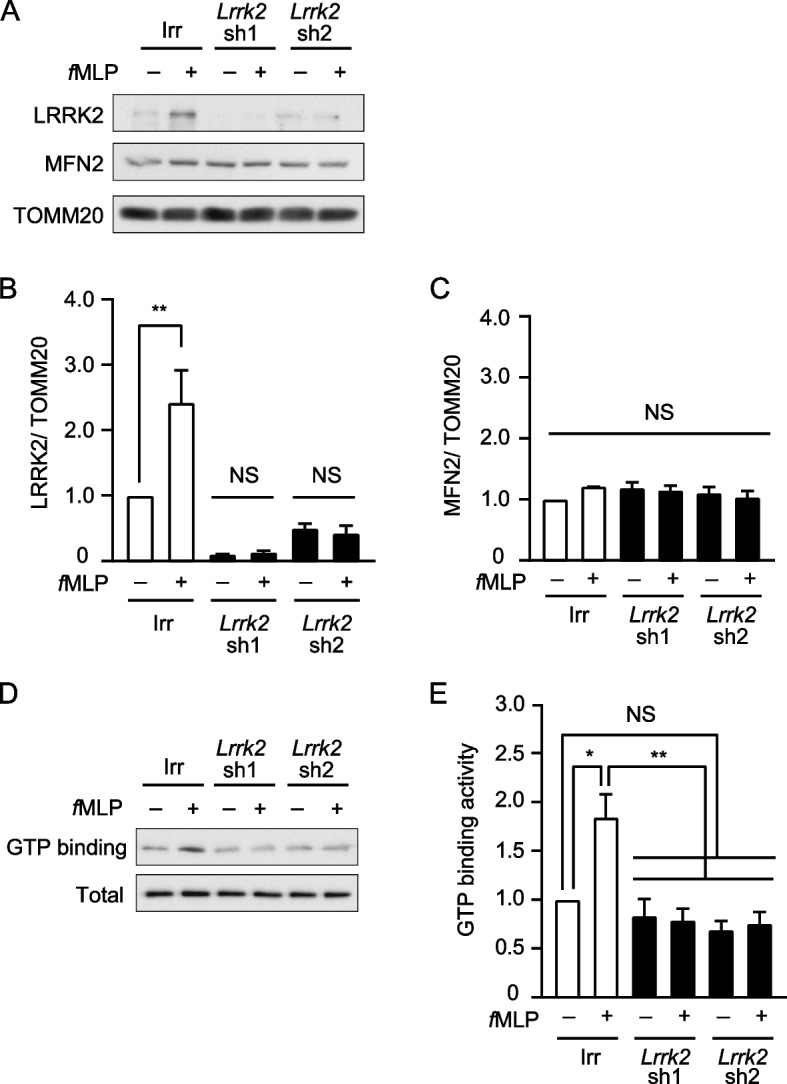
Mitochondrial fraction of LRRK2 and GTP binding activity of MFN2 were increased upon *f*MLP stimulation. **A**-**C** The mitochondrial fraction of LRRK2 was increased upon *f*MLP stimulation. dHL-60 cells expressing tetracycline-inducible *Lrrk2* shRNA or irrelevant (Irr) shRNA were cultured in 1.25% DMSO with 0.5 µg/ml doxycycline (Dox) for 3 days. The mitochondrial fraction of LRRK2 and MFN2 were measured with or without 1 µM *f*MLP for 5 min. **D**, **E** *Lrrk2* knockdown results in a decrease in MFN2 GTP-binding activity of dHL-60 cells. dHL-60 cells expressing tetracycline-inducible *Lrrk2* shRNA or irrelevant (Irr) shRNA were cultured in 1.25% DMSO with 0.5 µg/ml  Dox for 3 days. The GTP-binding activity of MFN2 was measured with or without 1 µM *f*MLP for 5 min. **A**, **D** Representative western blotting images. **B** The rate of LRRK2/TOMM20 in the mitochondrial fraction of Irr shRNA expressing dHL-60 cells without *f*MLP stimulation was taken as 1.0 (*n* = 3). ** *p* < 0.01 compared with Irr shRNA, *Lrrk2* sh1, or sh2 expressing dHL-60 cells without *f*MLP. **C** The rate of MFN2/TOMM20 in the mitochondrial fraction of Irr shRNA expressing dHL-60 cells without *f*MLP stimulation was taken as 1.0 (*n* = 3). NS, not significant. **E** GTP-binding activity of Irr shRNA expressing dHL-60 cells without *f*MLP was taken as 1.0 (*n* = 4). Error bars, SEM. ** *p* < 0.01 and * *p* < 0.05 compared with Irr shRNA expressing dHL-60 cells with or without 1 µM *f*MLP. NS, not significant

Because *Lrrk2* knockdown in dHL-60 cells reduced OXPHOS activity and suppressed mitochondrial morphology changes upon *f*MLP stimulation, we examined the effects of LRRK2 on the amount of MFN2 in the mitochondrial fraction. We found that the mitochondrial fraction of LRRK2 increased upon *f*MLP stimulation, whereas no notable changes were detected in the mitochondrial fraction of MFN2 upon *f*MLP stimulation (Fig. 4A-C). The effect of *Lrrk2* knockdown on the amount of MFN2 in the mitochondrial fraction was negligible (Fig. 4A, C).

We examined the GTP-binding activity of MFN2 following *f*MLP stimulation. The GTP-binding activity of MFN2 increased upon *f*MLP stimulation (Fig. 4D, E). In contrast, no increase in the GTP-binding activity of MFN2 was detected in *Lrrk2* knockdown dHL-60 cells upon *f*MLP stimulation (Fig. 4D, E). These results suggest that LRRK2 affects the GTP-binding activity of MFN2, rather than the amount of MFN2 in the mitochondrial fraction, upon *f*MLP stimulation.

### Inhibition of LRRK2 Kinase activity causes increased *f*MLP-induced chemotactic activity of dHL-60 cells

**Fig. 5:**
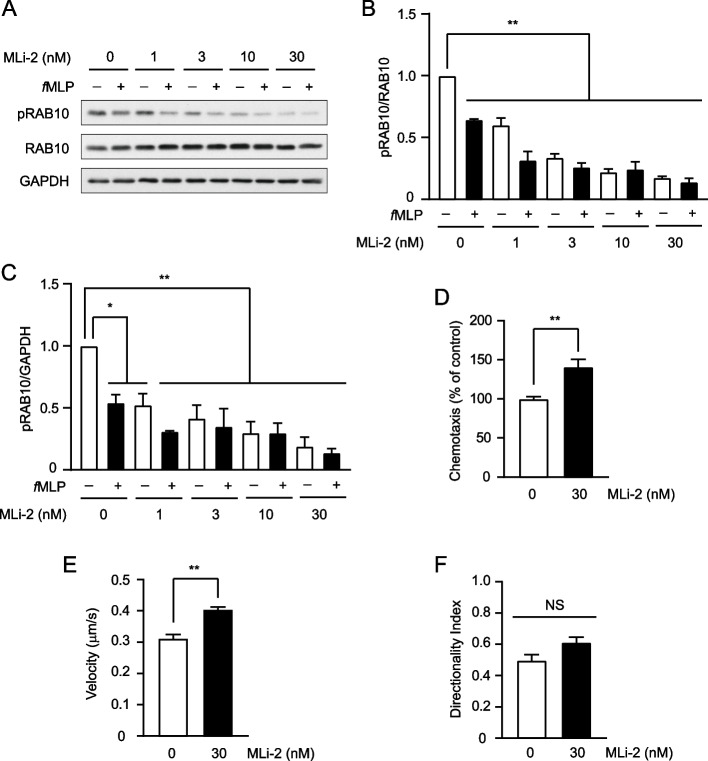
LRRK2 kinase inhibitor, MLi-2 activates chemotaxis of dHL-60 cells. **A**-**C** Effects of MLi-2 on kinase activity of LRRK2. dHL-60 cells were cultured in the presence of indicated concentrations of MLi-2 or 0.1% DMSO (vehicle) for 30 min. The cells were preincubated in HBSS containing 20 mM HEPES (pH 7.2) admixed with MLi-2 at indicated concentrations for 5 min at 37 °C and stimulated with or without 1 µM *f*MLP at 37 °C for 5 min. (**A**) Representative western blotting images. **B** The rate of Thr73 phosphorylation of RAB10 without *f*MLP/RAB10 without *f*MLP was taken as 1.0 (*n* = 3). (**C**) The rate of Thr73 phosphorylation of RAB10 without *f*MLP/GAPDH without *f*MLP was taken as 1.0 (*n* = 3). Error bars, SEM. ** *p* < 0.01 and * *p* < 0.05 compared to condition of 0.1% DMSO without *f*MLP. **D** Transwell chemotaxis assays of MLi-2 treated dHL-60 cells. dHL-60 cells were cultured with 30 nM MLi-2 or 0.1% DMSO (vehicle) for 30 min. The cell migration in response to 10 nM *f*MLP was measured with 30 nM MLi-2 or 0.1% DMSO for 120 min in transwell chambers (*n* = 6 wells). The activity of dHL-60 cells treated with 0.1% DMSO was taken as 100%. **E**, **F** Two-dimensional chemotaxis assays of MLi-2 treated dHL-60 cells. dHL-60 cells were cultured with 30 nM MLi-2 or 0.1% DMSO (vehicle) for 30 min. The cell migration in response to 10 nM *f*MLP was measured with 30 nM MLi-2 or 0.1% DMSO for 30 min in Dunn chambers (*n* > 16 cells). **E** Velocity. **F** Directionality index. ** *p* < 0.01 compared to the cells treated with 0.1% DMSO. NS, not significant

LRRK2 phosphorylates various proteins, including RAB10, RAB8 [29], and WAVE2 [30], and their phosphorylation affects membrane trafficking [31] and cytoskeleton remodeling [30], which are important for chemotaxis. The kinase activity of LRRK2 can be assessed based on the Thr73 phosphorylation level of RAB10 [32]. Therefore, we analyzed the Thr73 phosphorylation level of RAB10 to assess the kinase activity of LRRK2. The Thr73 phosphorylation level of RAB10 decreased upon *f*MLP stimulation, suggesting decreased kinase activity of LRRK2 (Fig. 5A-C). We also found that Ser935 phosphorylation of LRRK2 decreased upon *f*MLP stimulation (Fig. S4). Furthermore, we examined the effect of the LRRK2 kinase inhibitor MLi-2 on the chemotaxis of dHL-60 cells. We found that *f*MLP-induced chemotactic activity in dHL-60 cells was increased by MLi-2 treatment compared to that in untreated cells (Fig. 5D-F, S5, Additional files 6, 7). These results show that the kinase activity of LRRK2 negatively regulates chemotactic activity upon *f*MLP stimulation in dHL-60 cells.

## Discussion

In the present study, we found that *Lrrk2* knockout neutrophils and *Lrrk2* knockdown dHL-60 cells showed reduced chemotactic activity upon *f*MLP stimulation, and knockdown of *Lrrk2* reduced OXPHOS activity in dHL-60 cells. Furthermore, the amount of LRRK2 in the mitochondrial fraction increased upon *f*MLP stimulation, and LRRK2 affected the GTP-binding activity of MFN2. These results suggest that *f*MLP stimulation might translocate LRRK2 to the mitochondrial fraction and that LRRK2 might activate OXPHOS through MFN2 and cause chemotactic migration.

Chemotaxis requires a complex series of intracellular events such as cytoskeleton remodeling, membrane trafficking, and OXPHOS activation [3, 4, 33, 34]. LRRK2 is involved in membrane trafficking [31] and cytoskeleton remodeling [30]. We also observed reduced OXPHOS activity (Fig. 3A, B) and reduced GTP-binding activity of MFN2 (Fig. 4D, E) in *Lrrk2* knockdown dHL-60 cells. MFN2 is involved in OXPHOS activity [18] and actin cytoskeleton regulation [19] in dHL-60 cells. Therefore, the reduced chemotactic activity in *Lrrk2* knockout neutrophils and *Lrrk2* knockdown dHL-60 cells could be attributed to LRRK2, and MFN2-mediated effects.

We found that inhibition of LRRK2 kinase activity by MLi-2 in dHL-60 cells increased in *f*MLP-induced chemotactic activity (Fig. 5D-F). Kinase-dead LRRK2 (D1994A) in MEFs increased citrate synthase activity, OXPHOS activity, mitochondrial Ca^2+^ concentration, and the physical interaction between the ER and mitochondria compared to wild-type cells, although the facilitation of ATP production was undetectable [11]. It has been reported that a reduction in mitochondrial Ca^2+^ concentration due to the inhibition of the mitochondrial Ca^2+^ uniporter suppresses chemotactic activity in neutrophils [35]. Therefore, an increased mitochondrial Ca^2+^ concentration possibly increase the *f*MLP-induced chemotactic activity of dHL-60 cells. On the other hand, it was recently reported that inhibition of LRRK2 kinase activity by MLi-2 enhanced lysosomal degradative activity and increased the expression of multiple lysosomal hydrolases in induced pluripotent stem cell-derived macrophages [36]. Thus, the inhibition of LRRK2 kinase activity by MLi-2 may induce the expression of OXPHOS-associated and chemotaxis-associated proteins in dHL-60 cells. As described above, there are multiple possible mechanisms by which the inhibition of LRRK2 kinase activity by MLi-2 increases *f*MLP-induced chemotactic activity. Further studies are required to clarify how inhibition of LRRK2 kinase activity by MLi-2 facilitates *f*MLP-induced chemotactic activity.

In the present study, we found that the Ser935 phosphorylation of LRRK2 was reduced upon *f*MLP stimulation (Fig. S4). In general, protein phosphorylation is regulated by kinases and phosphatases. Ser935 of LRRK2 is phosphorylated by protein kinase A [37], an inhibitor of nuclear factor κB kinases [38], and casein kinase 1 α [39]. In contrast, Ser935 of LRRK2 is dephosphorylated by protein phosphatase 1 (PP1). Furthermore, it is reported that PP1α, which is expressed in neutrophils, preferentially interacts with LRRK2 [40]. Ser935 of LRRK2 is basally phosphorylated in cells, and LRRK2 has a long half-life [41]. Therefore, the short-term decrease in Ser935 phosphorylation of LRRK2 by *f*MLP stimulation may result from increased dephosphorylation of LRRK2 by phosphatases rather than decreased phosphorylation of LRRK2 by kinases. Phosphorylated Ser935 of LRRK2 is a 14–3-3 binding site [42], and the binding of 14–3-3 to LRRK2 affects its subcellular localization of LRRK2 [42]. We found that the mitochondrial fraction of LRRK2 increased upon *f*MLP stimulation (Fig. 4A, B). Reduced Ser935 phosphorylation of LRRK2 upon *f*MLP stimulation may affect the translocation of LRRK2 to the mitochondrial fraction by reducing 14–3-3 binding to LRRK2. On the other hand, Ser935 of LRRK2 was partially dephosphorylate, and the kinase activity of LRRK2 was partly decreased upon *f*MLP stimulation (Fig. 5A-C and Fig. S4). LRRK2 phosphorylate RAB proteins, including RAB5 and RAB10 [43]. RAB5 is present on early phagosomes, and regulate their fusion with early endosomes [44, 45]. LRRK2 forms a complex with the Wiskott–Aldrich syndrome protein-family verproline 2, and colocalizes with RAB5A during the fusion of phagosomes and early endosomes. Bone marrow-derived macrophages expressing LRRK2(G2019S) show increased phagocytic activity compared to wild-type macrophages [30]. Therefore, the kinase activity of LRRK2 is required, and may be partially required, for neutrophil and dHL-60 cell functions after *f*MLP stimulation.

We showed that the amount of LRRK2 in the mitochondrial fraction was increased upon *f*MLP stimulation, although the alteration of MFN2 level in the mitochondrial fraction was undetectable (Fig. 4A-C). The GTP-binding activity of MFN2 increased upon *f*MLP stimulation in Irr shRNA-expressing dHL-60 cells, whereas no notable changes were observed in *Lrrk2* knockdown dHL-60 cells (Fig. 4D, E). Therefore, it appears that the GTP-binding activity of MFN2 is related to the mitochondrial fraction of LRRK2. LRRK2 increases the GTP-binding activity of MFN1 in LRRK2 and MFN1 overexpressing cells [10]. Because MFN1 and MFN2 share approximately 80% sequence similarity, increased LRRK2 level in the mitochondrial fraction might increase the GTP-binding activity of MFN2 upon *f*MLP stimulation.

In addition to PD, LRRK2 has been reported to be associated with inflammatory bowel diseases (IBD) which is composed of two major subtypes, Crohn’s disease and ulcerative colitis, based on genome-wide association studies [46–49]. IBD is characterized by chronic proinflammatory responses by immune cells to viruses or bacteria in the gastrointestinal tract [50]. LRRK2 is involved in inflammatory signaling pathways, such as type I interferon signaling, and phagocytosis [12, 36, 45] and is expressed at higher levels in neutrophils than in other peripheral blood immune cells [32]. Therefore, LRRK2 mutations may exacerbate IBD by disruption of neutrophils function.

## Conclusions

This study showed that LRRK is involved in neutrophil chemotaxis and GTP binding activity of MFN2. Furthermore, it was suggested that the kinase activity of LRRK2 exerts a negative regulatory effect on chemotactic activity in dHL-60 cells upon *f*MLP stimulation. These results provide a better understanding of neutrophil chemotaxis and diseases involving LRRK2.

### Supplementary Information

**Additional file 1: Supplementary Figure S1.** Representative images with individual trajectories of neutrophil chemotaxis to *f*MLP. (A) wild-type neutrophils. (B) *Lrrk2* knockout neutrophils. Bar, 50 μm.

**Additional file 2: Supplementary Figure S2.** Representative images with individual trajectories of dHL-60 cells chemotaxis to *f*MLP. (A) irrelevant (Irr) shRNA expressing dHL-60 cells. (B) *Lrrk2* sh1 expressing dHL-60 cells. (C) *Lrrk2* sh2 expressing dHL-60 cells. Bar, 50 μm.

**Additional file 3: Supplementary Figure S3.** Transwell chemotaxis assays and two-dimensional chemotaxis assays of dHL-60 cells cultured without doxycycline. (A) dHL-60 cells expressing tetracycline-inducible*Lrrk2* shRNA or irrelevant (Irr) shRNA were cultured without doxycycline. dHL-60 cells migration in response to 10 nM *f*MLP measured for 120 min in transwell chambers (*n* = 6 wells). The activity of Irr shRNA expressing dHL-60 cells was taken as 100%. (B, C) dHL-60 cells migration in response to 10 nM *f*MLP measured for 30 min in Dunn chambers (n > 20 cells). (B) Velocity (C) Directionality index. Error bars, SEM. NS, not significant.

**Additional file 4: Supplementary Figure S4.** Ser935 phosphorylation status of LRRK2 upon *f*MLP stimulation. dHL-60 cells were preincubated in HBSS containing 20 mM HEPES (pH 7.2) for 5 min at 37°C, and then stimulated with or without 1 mM *f*MLP at 37 °C for 5 min. (A) Representative western blotting images. (B) The rate of Ser935 phosphorylation of LRRK2 without *f*MLP/total LRRK2 without *f*MLP was taken as 1.0 (*n* = 4). Error bars, SEM. ** *p* < 0.01 compared to the no *f*MLP condition.

**Additional file 5: Supplementary Figure S5.** Representative images with individual trajectories of dHL-60 cells chemotaxis to *f*MLP. (A) Vehicle-treated dHL-60 cells. (B) 30 nM MLi-2 treated dHL-60 cells. Bar, 50 μm.

**Additional file 6.** Representative movie of chemotactic migration of dHL-60 cells treated without MLi-2. Migration of dHL-60 cells treated without MLi-2 in response to 10 nM fMLP, and individual trajectories.

**Additional file 7.** Representative movie of chemotactic migration of dHL-60 cells treated with 30 nM MLi-2. Migration of dHL-60 cells treated with 30 nM MLi-2 in response to 10 nM fMLP, and individual trajectories.

## Data Availability

All data used in this study are available from the corresponding author on reasonable requests.

## Abbreviations

ER: Endoplasmic reticulum
*f*MLP: *N*-Formyl-Met-Leu-Phe peptide
LRRK2: Leucine-rich repeat kinase 2
MEFs: Mouse embryonic fibroblasts
MFN: Mitofusin
OCR: O_2_ consumption rates
OPA1: Optic atrophy 1
OXPHOS: Oxidative phosphorylation
